# Disrupted mother-fetus dyad risk in high-risk pregnancies: a Middle-Range Theory

**DOI:** 10.1590/0034-7167-2023-0464

**Published:** 2024-07-29

**Authors:** Ryanne Carolynne Marques Gomes Mendes, Gabrielle Pessôa da Silva, Jaqueline Galdino Albuquerque Perrelli, Cleide Maria Pontes, Lívia Maia Pascoal, Ana Luisa Brandão de Carvalho Lira, Marcos Venícios de Oliveira Lopes, Suzana de Oliveira Mangueira, Francisca Márcia Pereira Linhares

**Affiliations:** I Universidade Federal de Pernambuco. Recife, Pernambuco, Brazil; II Universidade Federal do Maranhão. Imperatriz, Maranhão, Brazil; III Universidade Federal do Ceará. Fortaleza, Ceará, Brazil

**Keywords:** Nursing Diagnosis, Pregnancy, High-Risk, Nursing Process, Maternal-Fetal Relations, Nursing Theory, Diagnóstico de Enfermería, Embarazo de Alto Riesgo, Proceso de Enfermería, Relaciones Materno-Fetales, Teoría de Enfermería

## Abstract

**Objectives:**

to develop and evaluate a Middle-Range Theory for the nursing diagnosis “Disrupted Mother-Fetus Dyad Risk” in high-risk pregnancies.

**Methods:**

this methodological study was conducted in two stages: theory development and evaluation. Dorothea Orem’s General Nursing Model was used as the theoretical-conceptual foundation. Evaluation was conducted using the Delphi method with seven judges, and consensus was achieved when the Content Validity Index of the evaluated items was ≥ 0.80.

**Results:**

the theory identified 20 elements of the nursing diagnosis “Disrupted Mother-Fetus Dyad Risk” (10 risk factors, 4 at-risk populations, and 6 associated conditions), 14 propositions, and 1 pictogram. After two rounds of evaluation, the theory was considered consistent, with consensus reached for all items, each achieving a Content Validity Index ≥ 0.80.

**Conclusions:**

the Middle-Range Theory included biopsychosocial factors explaining the nursing phenomenon “Disrupted Mother-Fetus Dyad Risk,” which aids in nurses’ diagnostic reasoning.

## RESUMEN

**Objetivos:**

desarrollar y evaluar una teoría de alcance intermedio para el diagnóstico de enfermería “Riesgo de Disrupción del Binomio Madre-Feto” en embarazos de alto riesgo.

**Métodos:**

este estudio metodológico se realizó en dos etapas: desarrollo y evaluación de la teoría. El modelo general de enfermería de Dorothea Orem se utilizó como base teórica-conceptual. La evaluación se realizó mediante el método Delphi con siete jueces, y se alcanzó el consenso cuando el Índice de Validez de Contenido de los ítems evaluados fue ≥ 0,80.

**Resultados:**

la teoría identificó 20 elementos del diagnóstico de enfermería “Riesgo de Disrupción del Binomio Madre-Feto” (10 factores de riesgo, 4 poblaciones en riesgo y 6 condiciones asociadas), 14 proposiciones y 1 pictograma. Después de dos rondas de evaluación, la teoría se consideró coherente, ya que se alcanzó el consenso en todos los ítems, cada uno logrando un Índice de Validez de Contenido ≥ 0,80.

**Conclusiones:**

la teoría de alcance intermedio incluyó factores biopsicosociales que explican el fenómeno de enfermería “Riesgo de Disrupción del Binomio Madre-Feto”, lo cual ayuda en el razonamiento diagnóstico de las enfermeras.

## INTRODUCTION

The mother-fetus dyad is characterized by the symbiotic relationship and maternal-fetal bond^(1)^. When this relationship or bond is interrupted, pregnant women are classified as high-risk and may experience pregnancy complications such as hemorrhage, placental abruption, uterine atrophy, premature membrane rupture, and others, which can lead to unfavorable outcomes^(2-3)^.

To prevent these outcomes, it is crucial for nurses to provide high-quality care to high-risk pregnant women by identifying nursing diagnoses^(4-5)^, such as “Disrupted Mother-Fetus Dyad Risk”, which is listed in the NANDA-International (NANDA-I) taxonomy under code 00209. This diagnosis is defined as “susceptibility to rupture of the symbiotic mother-fetus relationship due to comorbidity or pregnancy-related problems that could compromise health”^(6)^. Identifying this diagnosis allows for the planning and implementation of interventions that can reduce gestational risk and promote health^(5-6)^.

In identifying nursing diagnoses, developing Middle-Range Theory (MRT) provides insights into understanding the elements of the diagnosis and the causal relationships among them^(7)^. Moreover, evaluating these theories helps advance scientific knowledge in nursing by allowing the strengths and limitations of relationships between theoretical concepts to be assessed and determining whether new elements should be included or existing ones refined^(8)^.

Despite the growing interest in MRT studies^(9-11)^, research in this area remains limited, particularly regarding pregnancy complications. Additionally, these complications related to the disrupted mother-fetus dyad are often approached from a medical rather than a nursing perspective.

The development and evaluation of MRT can help identify the phenomenon of “Disrupted Mother-Fetus Dyad Risk” in high-risk pregnant women. This diagnosis was last reviewed in 2017 and has an evidence level of 2.1^(6)^, highlighting the need for studies that contribute to its update. This is an innovative and relevant proposal for nursing, especially due to its applicability in the profession.

There are published studies on concept analysis, content validation, and clinical validation of the nursing diagnosis “Disrupted Mother-Fetus Dyad Risk”^(1,12-13)^. However, a review of the scientific literature shows that no studies have yet addressed MRT for this phenomenon, making this study essential. It will contribute to the precise identification of the diagnosis and to implementing more appropriate nursing interventions, particularly those focused on promoting self-care and establishing the care that can be performed by high-risk pregnant women themselves or with nurses’ assistance.

This raises the following question: what is the assessment of the MRT developed for the nursing diagnosis “Disrupted Mother-Fetus Dyad Risk” in high-risk pregnant women?

## OBJECTIVES

To develop and evaluate a Middle-Range Theory for the nursing diagnosis “Disrupted Mother-Fetus Dyad Risk” in high-risk pregnant women.

## METHODS

### Ethical Considerations

The study was conducted in accordance with national and international ethical guidelines and was approved by the Research Ethics Committee of the Federal University of Pernambuco. Informed consent was obtained online from all participants involved in the study.

### Study Design, Period, and Location

This is a methodological study conducted from August 2021 to February 2022 at a public university in Pernambuco. The study was conducted in two stages: 1) Development of the MRT^(14)^ using the deductive theorizing approach and strategy, and 2) Evaluation of the MRT^(8)^.

### Population or Sample; Inclusion and Exclusion Criteria

The population consisted of Brazilian nurses who served as judges in evaluating the MRT. The sample included 7 judges who agreed to participate in the study. The Delphi technique recommends 5 to 20 judges^(8)^, thus justifying the number of participants in this study.

Inclusion criteria included nurses with a master’s or doctoral degree who met at least two of the following criteria: Experience in developing and/or evaluating nursing theories; Experience in high-risk pregnancy care; Supervision of research related to nursing theories; Scientific production related to nursing theories; Participation as speakers, lecturers, and/or attendees in courses or mini-courses on nursing theories; Nurses who did not respond to the invitation letter or who provided incomplete answers to the data collection instrument were excluded.

### Study Protocol

1) Development of Middle-Range Theory

The development stage involved six sub-steps: 1- Defining the construction approach; 2- Defining the theoretical-conceptual models to be analyzed; 3- Identifying the main concepts; 4- Developing a pictogram; 5- Constructing the propositions; and 6- Establishing causal relationships and practical evidence^(14)^.

The construction approach was based on the nursing phenomenon “Disrupted Mother-Fetus Dyad Risk”^(6)^ from the NANDA-I taxonomy in high-risk pregnancies. The theory was built upon elements from a previous study, an integrative review^(1)^, which identified two defining attributes of the “mother-fetus dyad” concept and 20 antecedents negatively affecting it (10 risk factors, 4 at-risk populations, and 6 associated conditions).

Dorothea Orem’s General Nursing Model, consisting of three interrelated theories-Self-Care, Self-Care Deficit, and Nursing Systems^(15)^ - was selected as the theoretical-conceptual model to be analyzed. This model served as the conceptual foundation for the MRT, enabling causal relationships between theoretical concepts and the formulation of propositions.

Regarding the main concepts, the MRT included the two attributes of the “mother-fetus dyad” concept, the 10 risk factors, the 4 at-risk populations, and the 6 associated conditions^(1,12)^. In addition, metaparadigm concepts (person, health, environment, and nursing) and the concepts from Dorothea Orem’s General Nursing Model, such as intrinsic and extrinsic conditioning factors, self-care requisites (universal, developmental, and health deviation), self-care deficit, and the support-education nursing system^(15)^, were used.

It’s worth noting that intrinsic and extrinsic conditioning factors were categorized as predispositional, precipitating, incapacitating, and reinforcing to establish a causal hierarchy among the concepts. Predispositional factors increase susceptibility to the phenomenon; incapacitating factors interfere with disease recovery and health promotion; precipitating factors start the causal chain; and reinforcing factors amplify the effect of a pre-existing clinical condition^(16)^.

A pictogram was created using elements of the MRT to visualize the phenomenon and causal relationships among the concepts. Clear and concise statements were developed during the construction of the propositions to relate the elements of the MRT.

These propositions served as a foundation for establishing causal relationships and practical evidence. The theoretical causal model for the nursing diagnosis “Disrupted Mother-Fetus Dyad Risk” was described, and clinical relationships were formed to enable logical clinical reasoning and judgment by nurses when caring for high-risk pregnant women. At this point, causal relationships between the phenomenon’s elements were established and later evaluated.

Causal relationships were established based on Dorothea Orem’s General Nursing Model principles^(15)^ and supported by scientific evidence on the “Disrupted Mother-Fetus Dyad Risk” in high-risk pregnant women, obtained through a literature review to facilitate coherent clinical reasoning and judgment.

2) Evaluation of the Middle-Range Theory

The evaluation stage of the MRT involved six sub-steps: 1. Selection of the type of Delphi method; 2. Identification and selection of judges; 3. Invitation to judges; 4. Categorization of judges; 5. Determination of the number of evaluation rounds; and 6. Definition of criteria for reaching consensus^(8)^.

When selecting the type of Delphi method, an approach known as normative evaluation was chosen to achieve consensus^(8)^. Identification and selection of judges were carried out through an advanced search on the Lattes Platform of the National Council for Scientific and Technological Development (CNPq). The keywords used were “Middle-Range Theory”, “Nursing Theory”, and “High-Risk Pregnancy”. Additionally, snowball sampling was employed, where one participant could recommend another.

Invitations were sent to judges via email, including the invitation letter and the Informed Consent Form (ICF) through the Google Forms platform. Those who agreed to participate in the study received evaluation instructions, the MRT, and a data collection instrument with variables for characterization (gender, region, degree, role, level of specialization, and scientific production related to nursing theory).

The instrument contained 18 items to evaluate the MRT, a subjective question, and spaces for suggestions. These items were adapted from questions used to evaluate MRTs^(17)^, such as: description of the theory’s purpose, theory type, theory origin, description of main concepts, description of propositions, context of use, definition of concepts, explanation of relationships, theory organization, theory diagram, theory clarification, concept foundation, prediction of outcomes, theory congruence, literature support, social relevance of the theory, cross-cultural relevance of the theory, and the theory’s contribution to nursing. The subjective question was: What are the implications for nursing regarding the implementation of the theory?

The categorization of judges was based on the principle of collective wisdom, considering educational background in nursing theory, professional experience in theory, metatheoretical knowledge and experience, dissemination of knowledge on nursing theories, and recognition of expertise in nursing theory. Thus, they were classified into the following expertise levels: beginner, advanced beginner, competent, proficient, and expert^(8)^.

The number of evaluation rounds was limited to a maximum of three to reach consensus. Items that did not reach consensus were not excluded, but the MRT was adjusted according to the judges’ suggestions, and the modified items were re-evaluated. In this study, two evaluation rounds were conducted.

The criteria for reaching consensus specified that the Content Validity Index (CVI) should be greater than or equal to 0.80. For each item, a five-point scale was used: 1 = Strongly disagree, 2 = Slightly agree, 3 = Partially agree, 4 = Strongly agree, and 5 = Fully agree.

### Analysis of Results and Statistics

The data were tabulated using double entry in Epi Info version 3.5.4 for database validation, then exported to Stata version 15.0.

The Content Validity Index (CVI) was calculated using the predictive diversity model, where the weight of each judge’s level of expertise was considered (beginner - weight 1; advanced beginner - weight 2; competent - weight 3; proficient - weight 4; expert - weight 5). The Shapiro-Wilk test was used to check for data normality. The mean or weighted median CVI was obtained, along with the corresponding 95% confidence intervals.

Consensus among the judges was achieved when the CVI was greater than or equal to 0.80. If the value was below 0.80, the MRT was adjusted according to the judges’ suggestions, and another evaluation round was conducted. Regarding the subjective question, the judges’ opinions were represented by the letter “J” and an Arabic number.

## RESULTS

The developed theory is predictive because it aims to establish causal relationships between concepts. It is titled “Disrupted Mother-Fetus Dyad Risk Theory in High-Risk Pregnancies”, a MRT that encompasses a set of less abstract concepts and ideas focused on a specific phenomenon.

The main concepts used in developing the MRT were the defining attributes, risk factors, at-risk populations, and conditions associated with the nursing diagnosis “Disrupted Mother-Fetus Dyad Risk” (Chart 1).

**Chart 1:** Primary concepts of the Middle-Range Theory for the nursing diagnosis “Disrupted Mother-Fetus Dyad Risk” in high-risk pregnancies, Recife, Pernambuco, Brazil, 2023

Defining Attributes
Symbiotic maternal-fetal relationship; Bond between mother and fetus
**Risk Factors**
Smoking; Inadequate/absent prenatal care; Illicit drug use; Alcohol abuse; Obesity; Unsatisfactory gestational weight gain; Smoking; Violence; Insufficient/absent partner support; Insufficient/absent social support.
**Risk Factors**
Pregnant women with low educational levels; Economically disadvantaged pregnant women; Extreme maternal age; Previous pregnancy with pre-eclampsia.
**Associated Conditions**
Gestational complications; Altered glucose metabolism; Compromised oxygen transfer to the fetus; Maternal diseases; Treatment regimen; Maternal conditions.

*Fonte: Gomes e colaboradores (2020)^(1)^ e Mendes e colaboradores (2021)^(12)^*

The primary concepts were theoretically and operationally defined in a previous study (12). An analogy was made between the antecedents and the intrinsic and extrinsic conditioning factors in Dorothea Orem’s General Nursing Model, which were divided into predispositional, precipitating, incapacitating, and reinforcing factors. The causal relationships between the concepts will be presented next (Chart 2).

**Chart 2:** Relationship between the primary and secondary concepts of the Middle-Range Theory for the nursing diagnosis “Disrupted Mother-Fetus Dyad Risk” in high-risk pregnancies, Recife, Pernambuco, Brazil, 2023

Intrinsic Conditioning Factors	Extrinsic Conditioning Factors
**• Predispositional**: Extreme maternal age; Alcohol abuse^*^; Illicit drug use^*^; Smoking^*^. **• Reinforcing**: Unsatisfactory gestational weight gain^**^; Overweight^*^; Obesity^*^; Previous pregnancy with pre-eclampsia^***^; Maternal conditions^***^. **• Precipitating**: Gestational complications^***^; Altered glucose metabolism^***^; Compromised oxygen transfer to the fetus^***^; Maternal diseases^***^.	**• Predispositional:** Violence^*^; Treatment regimen^***^. **• Incapacitating:** Inadequate/absent prenatal care+^**^; Insufficient/absent partner support^*^; Insufficient/absent social support^*^; Pregnant woman with a low educational level; Economically disadvantaged pregnant woman.
**• Metaparadigmatic Concepts: person, health, environment, and nursing.**	

* Caused by a deficit in universal self-care requisites;

** Caused by a deficit in developmental self-care requisites;

*** Caused by a deficit in health-deviation self-care requisites; +Corresponds to the absence/insufficiency of the nursing support-education system.

**Figure 1:**
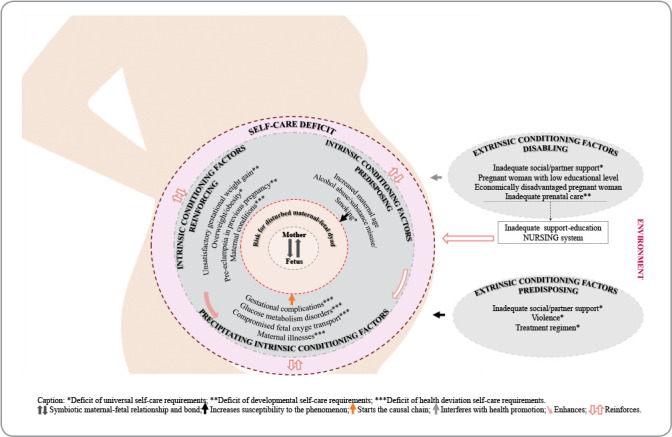
Pictogram of the Middle-Range Theory for the nursing diagnosis “Disrupted Mother-Fetus Dyad Risk” in high-risk pregnancies, Recife, Pernambuco, Brazil, 2023

The causal relationships between the elements of the MRT are also shown in the pictogram (Figure 1).

The pictogram shows the high-risk pregnant woman and four dotted circles. The central circle represents the symbiotic relationship and maternal-fetal bond, and the second circle represents the “Disrupted Mother-Fetus Dyad Risk” phenomenon. The third circle represents the interconnected intrinsic factors, while the fourth symbolizes the self-care deficit, which can affect the conditioning factors and, in turn, be affected by them, increasing susceptibility to the phenomenon. The outer section represents extrinsic conditioning factors, and the rectangle symbolizes the absence or insufficiency of the nursing support-education system, which can amplify the self-care deficit. Furthermore, the four metaparadigmatic concepts are visible: person (high-risk pregnant woman), health (preserved symbiotic maternal-fetal relationship), nursing (support-education system), and environment (social).

Fourteen propositions of the MRT were constructed as follows:

1. The health, life, and well-being of the mother-fetus dyad can be impacted by a self-care deficit. Thus, self-care is crucial for maintaining the dyad’s health, life, and well-being.
2. Disruption in the mother-fetus dyad can interrupt the symbiotic maternal-fetal relationship and the bond between mother and fetus, affecting both physiological exchanges and emotional interactions.
3. Antecedents that negatively affect the mother-fetus dyad can be classified as intrinsic or extrinsic conditioning factors. These factors can impact a high-risk pregnant woman’s ability and need for self-care, leading to a self-care deficit that may, in turn, exacerbate them.
4. Extreme maternal age, alcoholism, illicit drug use, and smoking are predispositional intrinsic conditioning factors. Violence, insufficient/absent social/partner support, and treatment regimen are predispositional extrinsic conditioning factors, increasing susceptibility to “Disrupted Mother-Fetus Dyad Risk”.
5. Unsatisfactory gestational weight gain, overweight, obesity, previous pregnancy with pre-eclampsia, and maternal conditions are reinforcing intrinsic conditioning factors because they amplify the effects of pre-existing clinical conditions in high-risk pregnancies.
6. Gestational complications, altered glucose metabolism, compromised oxygen transfer to the fetus, and maternal diseases can be intensified by reinforcing intrinsic conditioning factors and serve as precipitating factors, initiating the causal chain for “Disrupted Mother-Fetus Dyad Risk”.
7. Inadequate/absent prenatal care, insufficient/absent social/partner support, economically disadvantaged pregnant women, and pregnant women with low educational levels are incapacitating extrinsic conditioning factors because they interfere with recovery and health promotion for high-risk pregnant women.
8. The absence or insufficiency of the nursing support-education system (inadequate/absent prenatal care) can worsen the self-care deficit in high-risk pregnant women, disrupting intrinsic and extrinsic conditioning factors and promoting susceptibility to “Disrupted Mother-Fetus Dyad Risk”.
9. Intrinsic and extrinsic conditioning factors for “Disrupted Mother-Fetus Dyad Risk” can influence each other and disrupt the symbiotic maternal-fetal relationship.
10. The social environment of high-risk pregnant women (insufficient/absent partner or social support) can affect their health, increasing susceptibility to “Disrupted Mother-Fetus Dyad Risk”.
11. A deficit in universal self-care requisites among high-risk pregnant women can result in smoking, violence, insufficient/absent social or partner support, overweight, and obesity.
12. A deficit in developmental self-care requisites among high-risk pregnant women can lead to unsatisfactory gestational weight gain, pre-eclampsia in previous pregnancies, and inadequate/absent prenatal care.
13. A deficit in health-deviation self-care requisites can lead to maternal conditions, gestational complications, altered glucose metabolism, compromised oxygen transfer to the fetus, and maternal diseases.
14. Nursing should promote the support-education system to encourage self-care in high-risk pregnant women and foster health and social environmental balance to mitigate susceptibility to “Disrupted Mother-Fetus Dyad Risk”.

Establishing causal relationships and practical evidence was described in the discussion of this study, which was based on scientific research on “Disrupted Mother-Fetus Dyad Risk” in high-risk pregnant women and its elements.

During the evaluation of the MRT, it was observed that most judges were female (85.72%), lived in the Southeast and Northeast regions (42.86%), held a doctorate degree (57.14%), worked as educators (57.14%), and were classified as competent (57.14%). Regarding scientific production, 42.86% had evaluated a nursing theory, and the same percentage was found in studies on theory construction.

For the evaluated MRT items, the mean or median CVI weighted by level of expertise was calculated (Table 1).

**Table 1:** Evaluation of Middle-Range Theory items for the nursing diagnosis “Disrupted Mother-Fetus Dyad Risk” in high-risk pregnant women, Recife, Pernambuco, Brazil, 2023

Items	Shapiro-Wilk Test		Content Validity Index	
	W	*p* value	Weighted Mean or Median	95% CI
The theory's purpose (predictive) is described	0.869	0.182	0.85^‖^	0.787; 0.923
It is a MRT	0.537	<0.001	0.96^‖^	0.921; 0.998
The theory's origin is described	0.989	0.991	0.93^‖^	0.884; 0.975
The main concepts are described	0.537	<0.001	0.96^†^	0.902; 1.00
The main theoretical propositions are described	0.908	0.383	0.92^‖^	0.849; 0.990
The context for use is described	0.989	0.991	0.94^‖^	0.895; 0.984
The concepts are theoretically and operationally defined	0.537	<0.001	0.94^†^	0.871; 1.00
The relationships are explicit	0.980	0.959	0.81^‖^	0.732; 0.887
The theory is organized logically	0.908	0.383	0.92^‖^	0.849; 0.990
A model/diagram exists	^*^	^*^	1.0^‖^	^*^
The model/diagram contributes to clarifying the theory	0.537	<0.001	0.97^†^	0.935; 1.00
The concepts are well-founded	0.981	0.964	0.92^‖^	0.872; 0.967
Results or consequences are predicted	0.887	0.260	0.73^‖^	0.619; 0.619
The theory is congruent with current nursing standards	0.537	<0.001	0.98^†^	0.951; 1.00
It is supported by the literature	0.537	<0.001	0.98^†^	0.951; 1.00
The theory is socially relevant	0.537	<0.001	0.97^†^	0.935; 1.00
The theory has cross-cultural relevance	0.908	0.383	0.93^‖^	0.867; 0.992
The theory contributes to the discipline of nursing	^*^	^*^	1.0^‖^	^*^

‖ Weighted mean;

† Weighted median;

* Values with no variability.

Of the 18 items evaluated, 17 reached consensus in the first round. Only the item “Results or Consequences are Predicted” had a mean CVI < 0.80, which could have been due to a lack of clarity in some propositions.

The judges suggested that insufficient/absent social and partner support could be predispositional extrinsic conditioning factors. This suggestion was accepted because these factors make high-risk pregnant women more vulnerable to “Disrupted Mother-Fetus Dyad Risk”. Thus, adjustments were made to the propositions, which were sent for evaluation in the second round. Consensus was reached (CVI = 0.87, 95% CI [0.849; 0.890]).

Regarding the question, “What are the implications for nursing related to implementing the theory?” the judges stated that the MRT provides a comprehensive understanding of “Disrupted Mother-Fetus Dyad Risk”, enabling nurses to identify this nursing diagnosis and plan interventions for high-risk pregnant women. Additionally, they highlighted that the theory is concise and presents interconnected concepts coherently, which may facilitate the early detection of risk factors during pregnancy and contribute to reducing the disruption of the symbiotic maternal-fetal bond.

## DISCUSSION

The MRT developed in this study is predictive, as it establishes causal relationships between the listed concepts, which aids nurses’ critical thinking^(17)^. The theory focuses on the nursing diagnosis “Disrupted Mother-Fetus Dyad Risk” in high-risk pregnancies, identified when there is a predisposition to interrupt the maternal-fetal symbiosis due to pregnancy complications^(1)^.

A review of the scientific literature did not reveal any nursing theories addressing the phenomenon “Disrupted Mother-Fetus Dyad Risk”, underscoring the originality of this study and its contribution to nursing care for high-risk pregnancies. However, there is a theory that discusses the mother-fetus dyad: “The MRT for the Nursing Diagnosis of Excess Fluid Volume in Pregnant Women”^(18)^.

In this study, the theory was evaluated using the Delphi method, which, when combined with the collective wisdom approach, emphasizes anonymous communication between individuals with different levels of expertise^(8)^.

The “competent” level of expertise was the most prominent among the judges. This level refers to individuals with prior knowledge, critical thinking, and ideas on a specific topic^(19)^. Judges at the “advanced beginner” and “proficient” levels were also present. This variety is crucial for the evaluation process of the MRT, as it contributes to improving the theory^(8)^.

The causal relationships were assessed by the judges and can be seen in the propositions and the MRT pictogram. Several factors can lead to the phenomenon “Disrupted Mother-Fetus Dyad Risk,” and, if not avoided, can disrupt the maternal-fetal symbiotic relationship, compromising the well-being and health of the dyad^(1)^. These conditioning factors may be intrinsic or extrinsic to high-risk pregnancies and are interrelated. They can also affect self-care, which is crucial in high-risk pregnancies to prevent adverse outcomes^(4)^.

The intrinsic factor “extreme maternal age” is a predispositional factor for “Disrupted Mother-Fetus Dyad Risk”, as both adolescent pregnant women and those aged 35 or older are susceptible to this phenomenon (20-21). This is corroborated by the MRT for the nursing diagnosis “Excess Fluid Volume in Pregnant Women”, which lists extremes in reproductive age as a predispositional factor for pregnancy complications^(18)^.

Teen pregnancy is the leading cause of maternal mortality among women aged 10 to 19, and pregnancy complications are present in 18.7% of this population^(20)^. Women aged 35 and older also have a higher likelihood of various pregnancy complications^(21)^.

Alcohol abuse, illicit drug use, and smoking are also predispositional intrinsic factors because they relate to the health status of high-risk pregnant women. These conditions are present in approximately 5.1% of pregnant women and can result in adverse maternal-fetal outcomes^(22)^. Meanwhile, violence and the treatment regimen are predispositional extrinsic factors for “Disrupted Mother-Fetus Dyad Risk” because they make high-risk pregnant women susceptible to gestational complications, such as fetal malformations, spontaneous abortion, fetal death, and premature birth^(23-24)^.

In assessing these causal relationships, the judges suggested that insufficient/absent social and partner support should be considered predispositional extrinsic factors. This suggestion was accepted since high-risk pregnant women without financial, emotional, and/or instrumental support are more susceptible to rupturing the maternal-fetal symbiotic relationship^(14)^. Additionally, one item did not reach consensus in the first round because, according to the judges, some propositions needed rewriting. Propositions should be clear^(17)^, so a second evaluation round was necessary.

Regarding causal relationships, pregnant women who are overweight or obese, as well as those who have had pre-eclampsia in previous pregnancies, are at higher risk of developing pre-eclampsia, eclampsia, gestational diabetes, and hypertensive syndromes^(25-26)^. Unsatisfactory gestational weight gain, with a prevalence of 18.6% among high-risk pregnant women, reinforces gestational complications^(27)^. This aligns with the MRT, which also involves the mother-fetus dyad, indicating that vascular changes related to pregnancy-specific diseases are reinforcing factors for health issues during pregnancy^(18)^.

Gestational complications, altered glucose metabolism, compromised oxygen transfer to the fetus, and maternal diseases can initiate the causal chain because they trigger a series of adverse events for the mother-fetus dyad, making them precipitating intrinsic factors^(28)^.

Extrinsic factors like inadequate/absent prenatal care, insufficient/absent partner support, insufficient/absent social support, economically disadvantaged pregnant women, and pregnant women with low educational levels are considered incapacitating because they interfere with the recovery and health promotion of high-risk pregnant women^(29)^.

Inadequate or absent prenatal care is a potential contributor to the self-care deficit because it identifies factors that compromise the mother-fetus dyad and encourages self-care^(29)^. Self-care promotes activities that sustain the well-being of both the mother and fetus^(30-31)^. When prenatal care is absent or insufficient, promoting maternal-fetal health becomes ineffective^(32)^. By analogy to Dorothea Orem’s General Model, it’s evident there is a deficiency in the support-education nursing system^(10)^.

Low educational levels, low economic status, and insufficient/absent social or partner support also interfere with health promotion during pregnancy^(30,33)^. Studies show that poor education and low family income negatively impact pregnant women’s ability to practice self-care^(34-35)^. The absence of social and/or partner support is not only a predispositional factor but also an incapacitating one, as it tends to negatively impact the well-being of the mother-fetus dyad, affecting the social and psychological environment of high-risk pregnant women^(23,32-33)^.

Other aspects of the MRT evaluation include positive comments in response to the subjective question. The MRT represents a phenomenon of interest, providing a means to expand nursing knowledge and guide professional practice. This study advances nursing knowledge, as the constructed and evaluated MRT can be applied in teaching, research, and practice.

### Study limitations

The MRT developed may only apply to specific clinical conditions (high-risk pregnant women). Therefore, its generalization to low-risk pregnancies should be approached cautiously. Additionally, the selected theoretical model may have limited the causal relationships between concepts, and the judges’ subjectivity may have influenced the evaluation.

### Contributions to Nursing, Health, or Public Policy

This study contributes to nursing by supporting nurses’ diagnostic reasoning when caring for high-risk pregnant women, leading to interventions focused on promoting self-care and preventing gestational complications. Moreover, the MRT can enhance nursing scientific knowledge and update the nursing diagnosis “Disrupted Mother-Fetus Dyad Risk” in the NANDA-I taxonomy.

## CONCLUSIONS

The MRT was developed and evaluated, including biopsychosocial factors explaining the nursing phenomenon “Disrupted Mother-Fetus Dyad Risk”. The theory comprises 22 primary concepts that are elements of the diagnosis “Disrupted Mother-Fetus Dyad Risk” (2 attributes, 10 risk factors, 4 at-risk populations, and 6 associated conditions), 11 secondary concepts, and 14 propositions. Assumptions are not explicitly stated but can be inferred from the propositions since they are the premises that underpin the MRT.

These elements can help update the NANDA-I taxonomy, and the causal relationships established in the MRT can support the operationalization of the Nursing Process in high-risk pregnancy care.

The MRT was evaluated by the judges and achieved consensus on all items, demonstrating that the theory can be used in nursing education, research, and practice to identify the phenomenon “Disrupted Mother-Fetus Dyad Risk” in high-risk pregnancies and implement nursing interventions focused on promoting self-care.
