# Supplementary material for: Disrupted mother-fetus dyad risk in high-risk pregnancies: a Middle-Range Theory
Source: Rev Bras Enferm. 2024 Jul 29;77(3):e20230464. doi: 10.1590/0034-7167-2023-0464 (PMC11290727; doi:10.1590/0034-7167-2023-0464)
Supplement: Translation

# Translation: Risco de binômio mãe-feto perturbado em gestantes de alto risco: Teoria de Médio Alcance

**DOI:** 10.1590/0034-7167-2023-0464pt

**Authors:** Ryanne Carolynne Marques Gomes Mendes, Gabrielle Pessôa da Silva, Jaqueline Galdino Albuquerque Perrelli, Cleide Maria Pontes, Lívia Maia Pascoal, Ana Luisa Brandão de Carvalho Lira, Marcos Venícios de Oliveira Lopes, Suzana de Oliveira Mangueira, Francisca Márcia Pereira Linhares

**Affiliations:** I Universidade Federal de Pernambuco. Recife, Pernambuco, Brasil; II Universidade Federal do Maranhão. Imperatriz, Maranhão, Brasil; III Universidade Federal do Ceará. Fortaleza, Ceará, Brasil

**Keywords:** Diagnóstico de Enfermagem, Gestação de Alto Risco, Processo de Enfermagem, Relações Materno-Fetais, Teoria de enfermagem

## Abstract

**Objetivos:**

desenvolver e avaliar a Teoria de Médio Alcance para o diagnóstico de enfermagem “Risco de binômio mãe-feto perturbado” em gestantes de alto risco.

**Métodos:**

estudo metodológico desenvolvido em duas etapas: desenvolvimento e avaliação da teoria. Para a fundamentação teórico-conceitual, utilizou-se o Modelo Geral de Enfermagem de Dorothea Orem. A avaliação foi realizada por meio do método Delphi com 7 juízes, e o consenso foi obtido quando o Índice de Validade de Conteúdo dos itens avaliados foi ≥ 0,80.

**Resultados:**

a teoria apresentou 20 elementos do diagnóstico de enfermagem “Risco de binômio mãe-feto perturbado” (10 fatores de risco, 4 populações em risco e 6 condições associadas), 14 proposições e 1 pictograma. Além disso, após duas rodadas de avaliação, a teoria foi considerada consistente, pois houve consenso em todos os itens avaliados, que obtiveram um Índice de Validade de Conteúdo ≥ 0,80.

**Conclusões:**

a Teoria de Médio Alcance incluiu os fatores biopsicossociais que explicam o fenômeno de enfermagem “Risco de binômio mãe-feto perturbado”, o que contribui para o raciocínio diagnóstico do enfermeiro.

## INTRODUÇÃO

O binômio mãe-feto é caracterizado pela relação simbiótica e pelo vínculo materno-fetal^(1)^. Quando essa relação ou vínculo são interrompidos, as gestantes são classificadas como de alto risco e podem apresentar complicações gestacionais, como hemorragia, descolamento de placenta, atrofia uterina, ruptura prematura de membranas, entre outras, que podem resultar em desfechos desfavoráveis^(2-3)^.

Para evitar esses desfechos, é essencial que o enfermeiro ofereça assistência qualificada às gestantes de alto risco, identificando diagnósticos de enfermagem^(4-5)^, como o Risco de binômio mãe-feto perturbado, que consta na Taxonomia da *NANDA-International* (NANDA-I) - código 00209. Esse diagnóstico é definido como “susceptibilidade à ruptura da relação simbiótica mãe-feto em consequência de comorbidade ou problemas relacionados à gestação, que pode comprometer a saúde”^(6)^. A identificação desse diagnóstico permitirá o planejamento e a implementação de intervenções capazes de diminuir o risco gestacional e promover a saúde^(5-6)^.

No processo de identificação de diagnósticos de enfermagem, o desenvolvimento de Teoria de Médio Alcance (TMA) fornece subsídios para compreender os elementos do diagnóstico e a relação causal entre esses elementos^(7)^. Além disso, a avaliação dessas teorias contribui para o avanço do conhecimento científico da enfermagem, pois permite verificar as potencialidades e limitações das relações entre os conceitos teóricos, bem como identificar se é necessário incluir novos elementos na teoria ou aprimorar os já existentes^(8)^.

Apesar do crescente interesse em estudos sobre TMA^(9-11)^, ainda são incipientes as pesquisas dessa natureza, especialmente no contexto de complicações gestacionais. Além disso, frequentemente essas complicações relacionadas ao binômio mãe-feto perturbado são abordadas a partir de uma perspectiva médica, e não da enfermagem.

O desenvolvimento e a avaliação da TMA podem embasar a identificação do fenômeno Risco de binômio mãe-feto perturbado em gestantes de alto risco. Esse diagnóstico foi revisado pela última vez em 2017 e possui nível de evidência 2.1^(6)^, sendo necessário realizar estudos que contribuam para sua atualização. Essa é uma proposta inovadora e relevante para a área de enfermagem, principalmente devido à sua aplicabilidade na profissão.

Há estudos publicados sobre análise de conceito, validação de conteúdo e validação clínica do diagnóstico de enfermagem Risco de binômio mãe-feto perturbado^(1,12-13)^. Entretanto, após busca na literatura científica, observa-se que ainda não há estudos que abordem a TMA desse fenômeno, tornando necessário o desenvolvimento deste estudo, pois contribuirá para a identificação precisa do diagnóstico e para a implementação de intervenções de enfermagem mais adequadas, especialmente aquelas voltadas para a promoção do autocuidado, visando estabelecer os cuidados que podem ser realizados pelas próprias gestantes de alto risco ou com a ajuda de enfermeiros.

Assim, surge a seguinte questão: qual a avaliação da TMA desenvolvida para o diagnóstico de enfermagem “Risco de binômio mãe-feto perturbado” em gestantes de alto risco?

## OBJETIVOS

Desenvolver e avaliar a Teoria de Médio Alcance para o diagnóstico de enfermagem “Risco de binômio mãe-feto perturbado” em gestantes de alto risco.

## MÉTODOS

### Aspectos éticos

O estudo foi conduzido em conformidade com as diretrizes éticas nacionais e internacionais e aprovado pelo Comitê de Ética em Pesquisa da Universidade Federal de Pernambuco. O Termo de Consentimento Livre e Esclarecido foi obtido de todos os indivíduos envolvidos no estudo de forma online.

### Desenho, período e local de estudo

Este é um estudo metodológico, realizado entre agosto de 2021 e fevereiro de 2022, em uma universidade pública do estado de Pernambuco. Foi conduzido em duas etapas: 1) Desenvolvimento da TMA^(14)^ por meio da abordagem e estratégia de teorização dedutiva e 2) Avaliação da TMA^(8)^.

### População ou amostra; critérios de inclusão e exclusão

A população foi composta por enfermeiros brasileiros que atuaram como juízes na avaliação da TMA. A amostra consistiu de 7 juízes que aceitaram participar da pesquisa. A técnica Delphi recomenda a participação de 5 a 20 juízes^(8)^, justificando assim o número de participantes neste estudo.

Os critérios de inclusão foram: enfermeiros com título de mestre ou doutor que atendessem a pelo menos dois dos seguintes critérios: Experiência no desenvolvimento e/ou avaliação de teorias de enfermagem; Experiência na área de gestação de alto risco; Orientação de pesquisa relacionada a teorias de enfermagem; Produção científica relacionada a teorias de enfermagem; Participação como palestrantes, conferencistas e/ou ouvintes em cursos ou minicursos sobre teorias de enfermagem.

Foram excluídos os enfermeiros que não responderam à carta-convite e aqueles que responderam ao instrumento de coleta de dados de forma incompleta.

### Protocolo do estudo

1) Desenvolvimento da Teoria de Médio Alcance

A etapa de desenvolvimento ocorreu em seis subetapas: 1- Definição da abordagem de construção; 2- Definição dos modelos teórico-conceituais a serem analisados; 3- Definição dos conceitos principais; 4- Desenvolvimento de um pictograma; 5- Construção das proposições; e 6- Estabelecimento das relações de causalidade e evidências para a prática^(14)^.

A definição da abordagem de construção baseou-se no fenômeno de enfermagem da Taxonomia da NANDA-I, “Risco de binômio mãe-feto perturbado”^(6)^, em gestantes de alto risco. A construção da teoria foi ancorada nos elementos obtidos de uma pesquisa prévia, uma revisão integrativa^(1)^, que identificou dois atributos definidores do conceito “binômio mãe-feto” e 20 antecedentes que o afetam negativamente (10 fatores de risco, 4 populações em risco e 6 condições associadas).

Para a definição dos modelos teórico-conceituais a serem analisados, foi escolhido o Modelo Geral de Enfermagem de Dorothea Orem, que possui três teorias inter-relacionadas: do Autocuidado, do Déficit do Autocuidado e dos Sistemas de Enfermagem^(15)^. Esse modelo serviu como base teórico-conceitual para a TMA, permitindo a formulação de relações causais entre os conceitos da teoria, bem como a formulação das proposições.

Na definição dos conceitos principais, os principais conceitos da TMA foram os dois atributos do conceito “binômio mãe-feto”, os 10 fatores de risco, as 4 populações em risco e as 6 condições associadas do conceito^(1,12)^. Além disso, foram utilizados os conceitos do metaparadigma (pessoa, saúde, ambiente e enfermagem) e os conceitos do Modelo Geral de Enfermagem de Dorothea Orem, como fatores condicionantes intrínsecos, fatores condicionantes extrínsecos, requisitos de autocuidado (universal, desenvolvimental e de desvio da saúde), déficit de autocuidado e sistema de enfermagem apoio-educação^(15)^.

Ressalta-se que os fatores condicionantes intrínsecos e extrínsecos foram divididos em fatores predisponentes, precipitantes, incapacitantes e reforçadores, com o objetivo de estabelecer uma hierarquia causal entre os conceitos. Os fatores predisponentes aumentam a susceptibilidade ao fenômeno; os incapacitantes interferem na recuperação de doenças e na promoção da saúde; os precipitantes iniciam a cadeia causal; e os reforçadores amplificam o efeito de uma condição clínica preexistente^(16)^.

Para o desenvolvimento do pictograma, foi construído um esboço gráfico com os elementos da TMA, facilitando a compreensão do fenômeno e das relações causais entre os conceitos. Na construção das proposições, foram elaboradas afirmações claras e concisas para relacionar os elementos da TMA.

Essas proposições fundamentaram o estabelecimento das relações de causalidade e evidências para a prática, nas quais o modelo teórico causal para o diagnóstico de enfermagem “Risco de binômio mãe-feto perturbado” foi descrito, e foram desenvolvidas relações clínicas que permitem o raciocínio e julgamento clínico lógico do enfermeiro no cuidado à população de gestantes de alto risco. Nesse momento, foram estabelecidas as relações de causalidade entre os elementos que compõem o fenômeno, posteriormente avaliadas.

As relações causais foram estabelecidas com base nos princípios do Modelo Geral de Enfermagem de Dorothea Orem^(15)^ e fundamentadas em evidências científicas sobre o “Risco de perturbação no binômio mãe-feto” em gestantes de alto risco, obtidas por meio de revisão bibliográfica, visando facilitar a realização de um raciocínio e julgamento clínico coerentes.

2) Avaliação da Teoria de Médio Alcance

A etapa de avaliação da TMA ocorreu em seis subetapas: 1. Seleção do tipo de Delphi; 2. Localização e seleção dos juízes; 3. Convite aos juízes; 4. Categorização dos juízes; 5. Estabelecimento do número de rodadas de avaliação; e 6. Definição dos critérios para alcance do consenso^(8)^.

Na seleção do tipo de Delphi, foi escolhida uma abordagem que permitisse a obtenção do consenso, conhecida também como avaliação normativa^(8)^. A localização e seleção dos juízes ocorreram por meio de busca avançada na Plataforma Lattes do portal do Conselho Nacional de Desenvolvimento Científico e Tecnológico (CNPq). As palavras-chave utilizadas na busca foram “Teoria de Médio Alcance”, “Teoria de Enfermagem” e “Gestação de alto risco”. Além disso, foi empregada a abordagem de amostragem em bola de neve (*snowball sampling*), na qual um participante pode indicar outro.

Os convites aos juízes foram enviados por e-mail, contendo a carta-convite e o Termo de Consentimento Livre e Esclarecido (TCLE) por meio da plataforma *Google Forms*. Aqueles que aceitaram participar da pesquisa receberam as instruções para avaliação, a TMA e um instrumento de coleta de dados com variáveis para caracterização (sexo, região, titulação, atuação, nível de especialização, produção científica voltada para teoria de enfermagem).

Além disso, o instrumento continha 18 itens para avaliação da TMA, uma pergunta subjetiva e espaços para sugestões. Esses itens eram afirmativas adaptadas das questões para avaliação de TMA^(17)^, a saber: descrição da finalidade da teoria, tipo de teoria, origem da teoria, descrição dos principais conceitos, descrição das proposições, descrição do contexto de uso, definição dos conceitos, explicitação das relações, organização da teoria, diagrama da teoria, esclarecimento da teoria, fundamentação dos conceitos, previsão dos resultados, congruência da teoria, apoio da literatura, relevância social da teoria, relevância transcultural da teoria e contribuição da teoria para a enfermagem. A pergunta subjetiva foi: Quais são as implicações para a enfermagem relacionadas à implementação da teoria?

A categorização dos juízes foi baseada no princípio da sabedoria coletiva, levando em consideração a formação educacional em teoria de enfermagem, a experiência profissional na área da teoria, o conhecimento e experiência metateóricos, a disseminação do conhecimento produzido sobre teorias de enfermagem e o reconhecimento da especialização no tema das teorias de enfermagem. Dessa forma, foram classificados nos níveis de perícia: iniciante, iniciante avançado, competente, proficiente e perito^(8)^.

O estabelecimento do número de rodadas de avaliação foi feito com a escolha de um limite máximo de três rodadas para obtenção do consenso. Os itens que não alcançaram o consenso não foram excluídos, mas a TMA foi alterada conforme as sugestões dos juízes, e os itens modificados foram reavaliados. Neste estudo, foram realizadas duas rodadas de avaliação.

A definição dos critérios para alcance do consenso estabeleceu que o Índice de Validade de Conteúdo (IVC) deveria ser maior ou igual a 0,80 para obter consenso. Para cada item avaliado, foi utilizada uma escala de cinco pontos: 1 = Não concordo; 2 = Concordo pouco; 3 = Concordo parcialmente; 4 = Concordo muito; e 5 = Concordo totalmente.

### Análise dos resultados e estatística

Os dados foram tabulados por meio de dupla digitação no programa Epi Info, versão 3.5.4, para validação do banco de dados. Posteriormente, os dados foram exportados para o *software* Stata, versão 15.0.

O IVC foi calculado com base no modelo da diversidade preditiva, no qual o peso do nível de expertise dos juízes foi ponderado (iniciante - peso 1; iniciante avançado - peso 2; competente - peso 3; proficiente - peso 4; e perito - peso 5). O teste Shapiro-Wilk foi utilizado para verificar a normalidade dos dados. Obteve-se a média ou a mediana ponderada do IVC, com respectivos intervalos de confiança de 95%.

Houve consenso entre os juízes quando o IVC foi maior ou igual a 0,80. Quando o valor ficou abaixo de 0,80, foram feitas alterações na TMA conforme as sugestões dos juízes, e outra rodada de avaliação foi realizada. Quanto ao questionamento subjetivo, as opiniões dos juízes foram apresentadas, representadas pela letra “J” e um número arábico.

## RESULTADOS

A teoria desenvolvida é preditiva, pois busca estabelecer as relações causais entre conceitos, sendo intitulada “Teoria do Risco de binômio mãe-feto perturbado em gestantes de alto risco”. Trata-se de uma TMA, pois engloba um conjunto de conceitos menos abstratos e ideias focadas em um fenômeno.

Os principais conceitos utilizados no desenvolvimento da TMA foram os atributos definidores, os fatores de risco, as populações em risco e as condições associadas ao diagnóstico de enfermagem “Risco de binômio mãe-feto perturbado” (Quadro 1).

**Quadro 1:** Conceitos primários da Teoria de Médio Alcance para o diagnóstico de enfermagem Risco de binômio mãe-feto perturbado em gestantes de alto risco, Recife, Pernambuco, Brasil, 2023

Atributos definidores
Relação simbiótica materno-fetal; Vínculo entre a mãe e o feto.
**Fatores de risco**
Tabagismo; Cuidado pré-natal ausente/inadequado; Uso de drogas ilícitas; Abuso de álcool; Obesidade; Ganho de peso gestacional insatisfatório; Tabagismo; Violência; Apoio do parceiro ausente/insuficiente; Apoio social ausente/insuficiente.
**Populações em risco**
Gestante com baixo nível educacional; Gestante desfavorecida economicamente; Extremos de idade materna; Pré-eclâmpsia na gestação anterior.
**Condições associadas**
Complicações gestacionais; Alteração do metabolismo da glicose; Transferência de oxigênio ao feto comprometida; Doenças maternas; Regime de tratamento; Condições maternas.

*Fonte: Gomes e colaboradores (2020)^(1)^ e Mendes e colaboradores (2021)^(12)^*

Os conceitos primários foram definidos teoricamente e operacionalmente em estudo prévio^(12)^. E fez-se uma analogia entre os antecedentes e os conceitos fatores condicionantes intrínsecos e extrínsecos do Modelo Geral de Enfermagem de Dorothea Orem, os quais foram divididos em: predisponentes, precipitantes, incapacitantes e reforçadores. A seguir serão apresentadas as relações causais entre os conceitos (Quadro 2).

**Quadro 2:** Relação entre os conceitos principais e secundários da Teoria de Médio Alcance para o diagnóstico de enfermagem Risco de binômio mãe-feto perturbado em gestantes de alto risco, Recife, Pernambuco, Brasil, 2023

Fatores Condicionantes Intrínsecos	Fatores Condicionantes Extrínsecos
**• Predisponentes:** Extremos de idade materna; Abuso de álcool^^*^^; Uso de drogas ilícitas^^*^^; Tabagismo^^*^^. **• Reforçadores:** Ganho de peso gestacional insatisfatório^^**^^; Sobrepeso^^*^^; Obesidade^^*^^; Pré-eclâmpsia na gestação anterior^^***^^; Condições maternas^^***^^. **• Precipitantes:** Complicações gestacionais^^***^^; Alterações do metabolismo da glicose^^***^^; Transferência de oxigênio ao feto comprometida^^***^^; Doenças maternas^^***^^.	**• Predisponentes:** Violência^^*^^; Regime de tratamento^^***^^. **• Incapacitantes:** Cuidado pré-natal ausente/inadequado^+^**^^; Apoio do parceiro ausente/insuficiente^^*^^; Apoio social ausente/insuficiente^^*^^; Gestante com baixo nível educacional; Gestante desfavorecida economicamente.
**• Conceitos metaparadigmáticos:** pessoa, saúde, ambiente e enfermagem.	

^^*^^Ocasionado pelo déficit dos requisitos de autocuidado universal; ^^**^^Ocasionado pelo déficit dos requisitos de autocuidado desenvolvimental; ^^***^^Ocasionado pelo déficit dos requisitos de autocuidado de desvio de saúde;

+ Corresponde a ausência/insuficiência do sistema de enfermagem apoio-educação.

As relações causais entre os elementos da TMA também são observadas no pictograma (Figura 1)

**Figura 1:** Pictograma da Teoria de Médio Alcance para o diagnóstico de enfermagem Risco de binômio mãe-feto perturbado em gestantes de alto risco, Recife, Pernambuco, Brasil, 2023

O pictograma apresenta a gestante de alto risco e quatro círculos pontilhados: o círculo central representa a relação simbiótica e o vínculo materno-fetal; o segundo, o fenômeno “Risco de binômio mãe-feto perturbado”. O terceiro círculo representa os fatores intrínsecos interligados, enquanto o quarto simboliza o déficit de autocuidado, que pode afetar os fatores condicionantes e, ao mesmo tempo, ser afetado por eles, aumentando a susceptibilidade ao fenômeno. A parte externa representa os fatores condicionantes extrínsecos, e o retângulo simboliza a ausência ou insuficiência do sistema de enfermagem apoio-educação, o que pode potencializar o déficit de autocuidado. Além disso, é possível observar os quatro conceitos do metaparadigma: pessoa (gestante de alto risco), saúde (relação simbiótica materno-fetal preservada), enfermagem (sistema apoio-educação) e ambiente (social).

Foram construídas 14 proposições da TMA, a saber:

1. A saúde, a vida e o bem-estar do binômio mãe-feto podem ser afetados pelo déficit de autocuidado. Assim, o autocuidado é essencial para a manutenção da saúde, da vida e do bem-estar do binômio.
2. A perturbação do binômio mãe-feto pode interromper a relação simbiótica materno-fetal e o vínculo entre mãe e feto, pois pode afetar o intercâmbio fisiológico e as interações afetivo-emocionais.
3. Os antecedentes que afetam negativamente o binômio mãe-feto podem ser classificados como fatores condicionantes intrínsecos ou extrínsecos. Esses fatores podem afetar a capacidade e a necessidade da gestante de alto risco de cuidar de si mesma, ocasionando déficit de autocuidado, que pode, por sua vez, potencializá-los.
4. Extremidade da idade materna, alcoolismo, uso de drogas ilícitas e tabagismo são fatores condicionantes intrínsecos predisponentes. Violência, apoio social/do parceiro ausente/insuficiente e regime de tratamento são fatores condicionantes extrínsecos predisponentes, pois podem aumentar a susceptibilidade ao “Risco de binômio mãe-feto perturbado”.
5. Ganho de peso gestacional insatisfatório, sobrepeso, obesidade, pré-eclâmpsia em gestação anterior e condições maternas são fatores condicionantes intrínsecos reforçadores, pois podem amplificar o efeito das condições clínicas preexistentes na gestação de alto risco.
6. Complicações gestacionais, alteração do metabolismo da glicose, transferência comprometida de oxigênio ao feto e doenças maternas podem ser potencializadas pelos fatores condicionantes intrínsecos reforçadores e são precipitantes, pois podem iniciar a cadeia causal para o “Risco de binômio mãe-feto perturbado”.
7. Cuidado pré-natal ausente/inadequado, apoio social/do parceiro ausente/insuficiente, gestantes desfavorecidas economicamente e gestantes com baixo nível educacional são fatores condicionantes extrínsecos incapacitantes, pois podem interferir na recuperação e na promoção da saúde da gestante de alto risco.
8. A ausência/insuficiência do sistema de enfermagem apoio-educação (cuidado pré-natal ausente/inadequado) pode potencializar o déficit de autocuidado da gestante de alto risco, o que interfere nos fatores condicionantes intrínsecos e extrínsecos, promovendo a susceptibilidade ao “Risco de binômio mãe-feto perturbado”.
9. Os fatores condicionantes intrínsecos e extrínsecos para o “Risco de binômio mãe-feto perturbado” podem se influenciar mutuamente e interferir na relação simbiótica materno-fetal.
10. O ambiente social da gestante de alto risco (apoio do parceiro ou social ausente/insuficiente), quando afetado, pode impactar a saúde da gestante de alto risco e levar à susceptibilidade ao “Risco de binômio mãe-feto perturbado”.
11. O déficit dos requisitos de autocuidado universal nas gestantes de alto risco pode resultar no tabagismo, violência, apoio social e/ou do parceiro ausente/insuficiente, sobrepeso e obesidade.
12. O déficit dos requisitos de autocuidado desenvolvimental nas gestantes de alto risco pode causar ganho de peso gestacional insatisfatório, pré-eclâmpsia em gestações anteriores e cuidado pré-natal ausente/inadequado.
13. O déficit dos requisitos de autocuidado para desvio de saúde pode ocasionar condições maternas, complicações gestacionais, alterações no metabolismo da glicose, transferência comprometida de oxigênio ao feto e doenças maternas.
14. A enfermagem deve promover o sistema de apoio-educação, visando estimular o autocuidado nas gestantes de alto risco, além de promover a saúde e o equilíbrio do ambiente social para mitigar a susceptibilidade ao “Risco de binômio mãe-feto perturbado”.

O estabelecimento das relações de causalidade e evidências para a prática foi descrito na discussão deste estudo, fundamentado em pesquisas científicas sobre o “Risco de binômio mãe-feto perturbado” em gestantes de alto risco e seus elementos.

Na avaliação da TMA, observou-se que a maioria dos juízes era do sexo feminino (85,72%), residia nas regiões Sudeste e Nordeste (42,86%), tinha título de doutor (57,14%), trabalhava como docente (57,14%) e foi classificada como tendo nível de competência (57,14%). Quanto à produção científica, 42,86% avaliaram alguma teoria de enfermagem, e o mesmo percentual foi encontrado no desenvolvimento de estudos sobre a construção de teorias.

Em relação aos itens avaliados da TMA, foram calculadas a média ou a mediana do IVC ponderado pelo nível de expertise (Tabela 1).

**Tabela 1:** Avaliação dos itens da Teoria de Médio Alcance para o diagnóstico de enfermagem Risco de binômio mãe-feto perturbado em gestantes de alto risco, Recife, Pernambuco, Brasil, 2023

Itens	Teste Shapiro-Wilk		Índice de Validade de Conteúdo	
	W	Valor de *p*	Média ou mediana ponderada	IC95%
A finalidade (preditiva) da teoria está descrita	0,869	0,182	0,85^‖^	0,787; 0,923
É uma TMA	0,537	<0,001	0,96^‖^	0,921; 0,998
A origem da teoria está descrita	0,989	0,991	0,93^‖^	0,884; 0,975
Os principais conceitos estão descritos	0,537	<0,001	0,96^†^	0,902; 1,00
As principais proposições teóricas estão descritas	0,908	0,383	0,92^‖^	0,849; 0,990
O contexto para o uso está descrito	0,989	0,991	0,94^‖^	0,895; 0,984
Os conceitos estão definidos teórica e operacionalmente	0,537	<0,001	0,94^†^	0,871; 1,00
Os vínculos estão explícitos	0,980	0,959	0,81^‖^	0,732; 0,887
A teoria está organizada de forma lógica	0,908	0,383	0,92^‖^	0,849; 0,990
Existe um modelo/diagrama	^*^	^*^	1,0^‖^	^*^
O modelo/diagrama contribui para o esclarecimento da teoria	0,537	<0,001	0,97^†^	0,935; 1,00
Os conceitos estão bem fundamentados	0,981	0,964	0,92^‖^	0,872; 0,967
Os resultados ou as consequências estão previstos	0,887	0,260	0,73^‖^	0,619; 0,619
A teoria é congruente com os atuais padrões de enfermagem	0,537	<0,001	0,98^†^	0,951; 1,00
É apoiada pela literatura	0,537	<0,001	0,98^†^	0,951; 1,00
A teoria é socialmente relevante	0,537	<0,001	0,97^†^	0,935; 1,00
A teoria tem relevância transcultural	0,908	0,383	0,93^‖^	0,867; 0,992
A teoria contribui para a disciplina de enfermagem	^*^	^*^	1,0^‖^	^*^

‖ Média ponderada;

† Mediana ponderada;

* Valores que não apresentaram variabilidade.

Dos 18 itens avaliados, 17 atingiram consenso na primeira rodada de avaliação. Apenas o item “Os resultados ou as consequências estão previstos” apresentou uma média do IVC < 0,80, o que pode ter ocorrido devido à falta de clareza em algumas proposições.

Os juízes sugeriram que o apoio social e do parceiro ausente/insuficiente poderiam ser fatores condicionantes extrínsecos predisponentes. A sugestão foi aceita, pois esses fatores tornam a gestante de alto risco mais vulnerável ao “Risco de binômio mãe-feto perturbado”. Diante disso, foram realizados ajustes nas proposições, que foram enviadas para serem avaliadas na segunda rodada. O consenso foi alcançado (IVC = 0,87, IC 95% [0,849; 0,890]).

Quanto ao questionamento “Quais são as implicações para a enfermagem relacionadas à implementação da teoria?”, os juízes afirmaram que a TMA permite uma compreensão abrangente do “Risco de binômio mãe-feto perturbado”, capacitando os enfermeiros a identificar esse diagnóstico de enfermagem e planejar intervenções para gestantes de alto risco. Além disso, destacaram que a teoria é parcimoniosa e apresenta conceitos interligados de forma coerente, o que pode facilitar a detecção precoce de fatores de risco na gestação e contribuir para a redução da ruptura da simbiose e do vínculo materno-fetal.

## DISCUSSÃO

A TMA construída neste estudo é preditiva, pois estabelece relações causais entre os conceitos elencados, o que auxilia o pensamento crítico do enfermeiro^(17)^. A teoria concentra-se no diagnóstico de enfermagem “Risco de perturbação no binômio mãe-feto em gestantes de alto risco”, identificado quando há uma predisposição à interrupção da simbiose materno-fetal devido a complicações gestacionais^(1)^.

Uma busca na literatura científica não revelou a existência de teorias de enfermagem que abordem o fenômeno “Risco de binômio mãe-feto perturbado”, destacando a originalidade deste estudo e sua contribuição para a assistência de enfermagem a gestantes de alto risco. No entanto, existe uma teoria que discute o binômio mãe-feto: a “ TMA para o diagnóstico de enfermagem de Volume de Líquidos Excessivo em gestantes”^(18)^.

Neste estudo, a teoria foi avaliada por meio do método Delphi que, associado à abordagem da sabedoria coletiva, é caracterizado por enfatizar a comunicação anônima entre indivíduos com diferentes níveis de expertise^(8)^.

O nível de expertise “competente” foi o que mais se destacou entre os juízes. Esse nível se refere a indivíduos que possuem conhecimento prévio, raciocínio crítico e ideias a respeito de um determinado assunto^(19)^. Juízes nos níveis “iniciante avançado” e “proficiente” também estiveram presentes. Essa variedade é essencial para o processo de avaliação da TMA, pois contribui para o aperfeiçoamento da teoria^(8)^.

As relações causais foram avaliadas pelos juízes e podem ser observadas nas proposições e no pictograma da TMA. Vários fatores podem levar ao fenômeno “Risco de binômio mãe-feto perturbado”, e, quando não evitados, podem interromper a relação simbiótica materno-fetal, comprometendo o bem-estar e a saúde desse binômio ^(1)^. Esses fatores condicionantes podem ser intrínsecos ou extrínsecos à gestação de alto risco e estão inter-relacionados. Além disso, podem comprometer o autocuidado, que, no contexto da gestação de alto risco, é essencial para prevenir desfechos desfavoráveis^(4)^.

O fator intrínseco “extremos de idade materna” é predisponente ao “Risco de binômio mãe-feto perturbado”, pois tanto gestantes adolescentes quanto aquelas com 35 anos ou mais são suscetíveis a esse fenômeno^(20-21)^. Isso é corroborado pela TMA do diagnóstico de enfermagem “Volume de líquidos excessivos em gestantes”, que indica os extremos de idade reprodutiva como um fator predisponente para a ocorrência de problemas gestacionais^(18)^.

A gravidez na adolescência é a principal causa de mortalidade materna em mulheres entre 10 e 19 anos, e complicações gestacionais estão presentes em 18,7% dessa população^(20)^. Mulheres com 35 anos ou mais também apresentam maior probabilidade de diversas complicações gestacionais^(21)^.

O abuso de álcool, o uso de drogas ilícitas e o tabagismo também são fatores intrínsecos predisponentes, pois estão relacionados ao estado de saúde das gestantes de alto risco. Essas condições estão presentes em aproximadamente 5,1% das gestantes e podem ocasionar resultados materno-fetais adversos^(22)^. Enquanto isso, a violência e o regime de tratamento são fatores extrínsecos predisponentes ao “Risco de binômio mãe-feto perturbado”, pois tornam as gestantes de alto risco suscetíveis a complicações gestacionais, como malformações fetais, aborto espontâneo, óbito fetal e parto prematuro^(23-24)^.

Na avaliação dessas relações causais, os juízes sugeriram que o apoio social e o apoio do parceiro ausente/insuficiente fossem fatores extrínsecos predisponentes. A sugestão foi acatada, visto que, quando a gestante de alto risco não recebe suporte financeiro, emocional e/ou instrumental, ela se torna mais suscetível à ruptura da relação simbiótica materno-fetal^(14)^. Além disso, um item não alcançou consenso na primeira rodada de avaliação, pois, segundo os juízes, algumas proposições precisavam ser reescritas. Sabe-se que as proposições devem ser claras^(17)^, e por isso foi necessária uma segunda rodada de avaliação.

Ainda sobre as relações causais, gestantes com sobrepeso e obesidade, bem como aquelas que tiveram pré-eclâmpsia em gestações anteriores, têm um risco maior de desenvolver pré-eclâmpsia, eclâmpsia, diabetes gestacional e síndromes hipertensivas^(25-26)^. O ganho de peso gestacional insatisfatório, com uma prevalência de 18,6% entre gestantes de alto risco, reforça as complicações gestacionais^(27)^. Esses dados se assemelham aos da TMA que também envolve o binômio mãe-feto, indicando que alterações vasculares relacionadas a doenças específicas da gestação são fatores reforçadores para problemas de saúde durante o período gestacional^(18)^.

Complicações gestacionais, alteração no metabolismo da glicose, transferência comprometida de oxigênio ao feto e doenças maternas podem iniciar a cadeia causal, já que desencadeiam uma série de eventos desfavoráveis ao binômio mãe-feto, sendo, portanto, fatores intrínsecos precipitantes^(28)^.

Os fatores extrínsecos, como cuidado pré-natal ausente/inadequado, apoio do parceiro ausente/insuficiente, apoio social ausente/insuficiente, gestantes desfavorecidas economicamente e gestantes com baixo nível educacional, são considerados incapacitantes, pois interferem na recuperação e promoção da saúde da gestante de alto risco^(29)^.

O cuidado pré-natal ausente/inadequado é um potencializador do déficit de autocuidado, pois essa assistência contribui para a identificação dos fatores que comprometem o binômio mãe-feto e para o estímulo ao autocuidado^(29)^. O autocuidado favorece o desenvolvimento de atividades que mantêm o bem-estar tanto da mãe quanto do feto^(30-31)^. Quando o cuidado pré-natal é inexistente ou insuficiente, a promoção da saúde materno-fetal é ineficaz^(32)^. Por analogia com o Modelo Geral de Dorothea Orem, pode-se dizer que há insuficiência no sistema de enfermagem apoio-educação^(10)^.

O baixo nível educacional, o baixo nível econômico e o apoio social ou do parceiro ausente/insuficiente também interferem na promoção da saúde durante o período gestacional^(30,33)^. Estudos indicam que um nível precário de escolaridade, combinado com baixa renda familiar, são fatores negativos para a prática do autocuidado das gestantes^(34-35)^. A ausência do apoio social e/ou do parceiro, além de ser um fator predisponente, também é incapacitante, pois tende a influenciar negativamente o bem-estar do binômio mãe-feto, afetando o ambiente social e psicológico da gestante de alto risco^(23,32-33)^.

Outros aspectos da avaliação da TMA incluem comentários positivos em resposta à questão subjetiva. Observa-se que a TMA representa um fenômeno de interesse, sendo um meio para a construção do corpo do conhecimento em enfermagem e capaz de direcionar a prática profissional do enfermeiro. Assim, este estudo avança no conhecimento da enfermagem, pois a TMA construída e avaliada pode ser aplicada no ensino, na pesquisa e na prática.

### Limitações do estudo

A TMA desenvolvida pode ser aplicável apenas a condições clínicas específicas (gestantes de alto risco). Portanto, sua generalização para gestantes de risco habitual deve ser feita com cautela. Além disso, a escolha do modelo teórico pode ter limitado as relações causais entre os conceitos, e a subjetividade dos juízes pode ter interferido na avaliação.

### Contribuições para a área da Enfermagem, saúde ou política pública

Este estudo contribui para a área de enfermagem, pois pode apoiar o raciocínio diagnóstico do enfermeiro na assistência a gestantes de alto risco e, consequentemente, na implementação de intervenções focadas na promoção do autocuidado e na prevenção de agravos gestacionais. Ademais, a TMA pode contribuir para o conhecimento científico da enfermagem e para a atualização do diagnóstico de enfermagem “Risco de binômio mãe-feto perturbado” na taxonomia da NANDA-I.

## CONCLUSÕES

A TMA foi desenvolvida e avaliada, incluindo os fatores biopsicossociais que explicam o fenômeno de enfermagem “Risco de binômio mãe-feto perturbado”. A teoria contém 22 conceitos primários, que são os elementos do diagnóstico “Risco de binômio mãe-feto perturbado” (2 atributos, 10 fatores de risco, 4 populações em risco e 6 condições associadas), 11 conceitos secundários e 14 proposições. Os pressupostos não estão explicitamente declarados, mas podem ser inferidos das proposições, já que são as suposições que sustentam a TMA.

Esses elementos podem contribuir para a atualização da Taxonomia da NANDA-I, e as relações causais estabelecidas na TMA podem apoiar a operacionalização do Processo de Enfermagem na assistência a gestantes de alto risco.

A TMA foi avaliada pelos juízes e alcançou consenso em todos os itens, demonstrando que a teoria pode ser utilizada no ensino, na pesquisa e na prática de enfermagem, para identificar o fenômeno “Risco de binômio mãe-feto perturbado” em gestantes de alto risco e implementar intervenções de enfermagem focadas na promoção do autocuidado.
